# Insights from the protein interaction Universe of the multifunctional “Goldilocks” kinase DYRK1A

**DOI:** 10.3389/fcell.2023.1277537

**Published:** 2023-10-12

**Authors:** Varsha Ananthapadmanabhan, Kathryn H. Shows, Amanda J. Dickinson, Larisa Litovchick

**Affiliations:** ^1^ Department of Internal Medicine, Division of Hematology, Oncology and Palliative Care, Virginia Commonwealth University, Richmond, VA, United States; ^2^ Department of Biology, Virginia State University, Petersburg, VA, United States; ^3^ Department of Biology, Virginia Commonwealth University, Richmond, VA, United States; ^4^ Massey Cancer Center, Richmond, VA, United States

**Keywords:** DCAF7, RNF169, GLCCI1, FAM117B, FAM53C, TROAP, proteomic analysis, development

## Abstract

Human Dual specificity tyrosine (Y)-Regulated Kinase 1A (DYRK1A) is encoded by a dosage-dependent gene located in the Down syndrome critical region of human chromosome 21. The known substrates of DYRK1A include proteins involved in transcription, cell cycle control, DNA repair and other processes. However, the function and regulation of this kinase is not fully understood, and the current knowledge does not fully explain the dosage-dependent function of this kinase. Several recent proteomic studies identified DYRK1A interacting proteins in several human cell lines. Interestingly, several of known protein substrates of DYRK1A were undetectable in these studies, likely due to a transient nature of the kinase-substrate interaction. It is possible that the stronger-binding DYRK1A interacting proteins, many of which are poorly characterized, are involved in regulatory functions by recruiting DYRK1A to the specific subcellular compartments or distinct signaling pathways. Better understanding of these DYRK1A-interacting proteins could help to decode the cellular processes regulated by this important protein kinase during embryonic development and in the adult organism. Here, we review the current knowledge of the biochemical and functional characterization of the DYRK1A protein-protein interaction network and discuss its involvement in human disease.

## 1 Introduction

Protein kinases catalyze the transfer of a phosphate from ATP on to the side chains of their substrates. This reaction results in a negative charge at the phosphorylation site, which often triggers changes in the function of these substrate proteins. Since the activity of protein kinases could also be subject to regulation, they can serve as molecular switches that relay intracellular signals. Protein kinases can be regulated through phosphorylation mediated by an upstream kinase, or by conformational changes caused by interaction with other proteins or ligands (Huse and Kuriyan, 2002; Pellicena and Kuriyan, 2006). Many kinases exist in a poised, inactive state until they are activated in response to environmental signals. Upon activation, a rapid cascade of events occurs that will then determine cellular responses, such as cell growth and proliferation under favorable conditions, or cell cycle arrest and even death in the event of environmental stress. Other kinases can be active at a baseline level but are restrained by phosphorylation or the binding of a protein inhibitor. If these regulatory mechanisms are disrupted by mutations, kinases can assume a constitutively active or inactive state, resulting in dysregulation of the downstream signaling and detrimental effects such as cancer. Given the undeniable potential of protein kinases as therapeutic targets in human disease, these enzymes remained in the focus of extensive research for decades, especially since a deeper insight into their structure was made possible by the crystallography studies of protein kinase A (PKA), cyclin dependent kinase 2 (CDK2), and other kinases (Knighton et al., 1991; De Bondt et al., 1993).

Dual specificity Tyrosine (Y) Regulated Kinase (DYRK1A) is an attractive target for therapeutic drug development due to its roles during embryonic development and in major human pathological conditions including Down syndrome (DS), Alzheimer’s disease (AD), and cancer, which are summarized in these excellent recent reviews (Atas-Ozcan et al., 2021; Kumar et al., 2021; Laham et al., 2021; Deboever et al., 2022; Rammohan et al., 2022; Santos-Duran and Barreiro-Iglesias, 2022). Indeed, several promising DYRK1A inhibitor drugs are currently in development for treatment of neurodegeneration, diabetes, and cancer (Pallavicini et al., 2019; Scavuzzo and Borowiak, 2020; Kumar et al., 2021; Liu T. et al., 2022; Guo et al., 2022; de Souza et al., 2023). However, our understanding of the function of DYRK1A is far from complete. DYRK1A has been shown to phosphorylate multiple protein substrates involved in regulation of cell cycle, transcription, DNA damage repair, apoptosis and other cellular processes that could not be integrated into a distinct pathway (Fernandez-Martinez et al., 2015). One the other hand, the mechanisms by which DYRK1A is regulated are not as well characterized, making it difficult to predict the environmental stimuli that could influence DYRK1A activity in the cells. Recent proteomic studies from several groups identified DYRK1A interacting proteins in human cells (Varjosalo et al., 2013; Guard et al., 2019; Menon et al., 2019; Roewenstrunk et al., 2019). These studies revealed a surprisingly large number of binding partners, and only a few of those proteins have been previously associated with DYRK1A. Moreover, such interacting proteins appear to represent a variety of cellular compartments, biological processes and signaling pathways, further adding to a complexity of the functional network that involves DYRK1A. For example, these DYRK1A interactome studies have already resulted in the discovery of a novel role of DYRK1A in DNA repair (Guard et al., 2019; Menon et al., 2019; Roewenstrunk et al., 2019). Therefore, there is no doubt that continued investigation of the DYRK1A interactome will allow us to uncover additional pieces of the DYRK1A puzzle, leading to a better understanding of this important protein kinase. In this review, we will summarize the recent advances in DYRK1A research in the context of both normal and diseased states.

## 2 Distinct mechanism of activation and substrate specificity of DYRK1A

### 2.1 Classification and localization of DYRK1A

**FIGURE 1:**
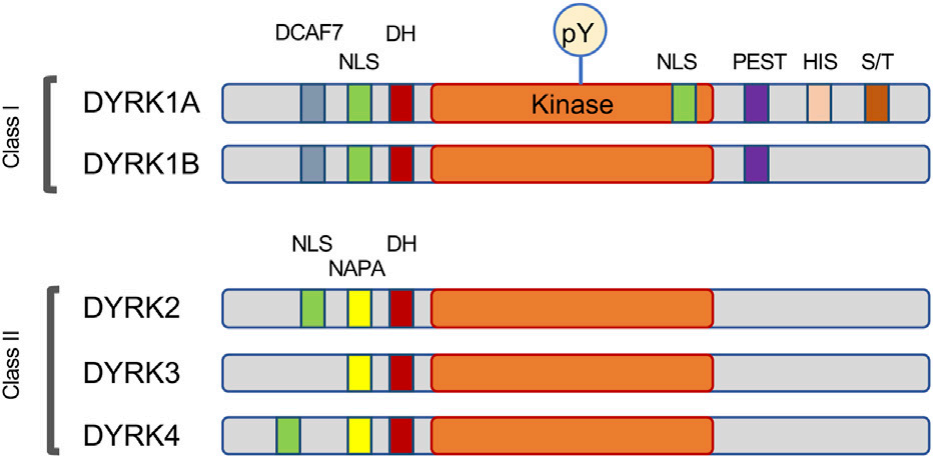
Schema of the motifs found in DYRK family of protein kinases. This family is characterized by autophosphorylation of a key tyrosine (pY) residue within the activation loop, and by the presence of the DYRK-homology box (DH). Most DYRK proteins have a recognizable nuclear localization signal (NLS). The Class I kinases DYRK1A and DYRK1B share a DCAF7-binding domain and the motif rich in proline, glutamic acid, serine, and threonine residues (PEST), while the Class II kinases are characterized by the N-terminal autophosphorylation accessory region (NAPA). DYRK1A also contains several unique motifs, including a second NLS within the kinase domain, a histidine-rich domain (HIS) and a regulatory serine and threonine-rich motif (S/T).

DYRK1A belongs to the CMGC group of eukaryotic protein kinases that includes cyclin-dependent kinases or CDKs, mitogen-activated protein kinases or MAPKs, glycogen synthase kinases or GSKs, and CDC-like kinases or CLKs (Hanks and Hunter, 1995; Kannan and Neuwald, 2004; Aranda et al., 2011). DYRK1A is one of five mammalian DYRK kinases, and along with closely related DYRK1B it was initially classified as nuclear, or Class I DYRK, while related DYRK2, DYRK3 and DYRK4 were classified as the cytosolic Class II kinases (Becker et al., 1998). However, knowledge of subcellular distribution of DYRK proteins at the endogenous levels showed that this classification is not accurate, and the distinction of Class I and Class II DYRKs can be based on their characteristic sequence motifs (Figure 1). Indeed, studies of endogenous proteins showed that DYRK1A accumulates in the nuclear speckles under overexpressed conditions, but endogenous DYRK1A localizes both in the nucleus and cytoplasm in multiple cell types (Alvarez et al., 2003; Marti et al., 2003; Wegiel et al., 2004; Hammerle et al., 2008; Guard et al., 2019; Menon et al., 2019; Roewenstrunk et al., 2019). Both DYRK1A and DYRK1B contain an N-terminal bi-partite nuclear localization signal (NLS), while DYRK1A also has a second complex NLS within its catalytic kinase domain (Becker et al., 1998). The DYRK1A kinase domain is followed by a histidine-rich domain that targets this protein to the nuclear speckles where it may co-localize with splicing machinery (Becker et al., 1998; Alvarez et al., 2003). Interestingly, histidine-rich domain of DYRK1A has been shown to promote the formation of phase-separated liquid droplets that contain RNA Polymerase II and other factors essential for transcription (Lu et al., 2018). Recent study also demonstrated a regulatory role of this domain by histidine polyphosphate modification that inhibits DYRK1A kinase activity (Neville et al., 2023). Unlike other DYRK family members, DYRK1A is ubiquitously expressed in the tissues of both the adult and fetal origin (Becker and Joost, 1999; Guimera et al., 1999; Leder et al., 1999; Okui et al., 1999; Zhang et al., 2005; Mercer and Friedman, 2006; Sacher et al., 2007; Hammerle et al., 2008; Soppa and Becker, 2015).

### 2.2 Autophosphorylation and substrate recognition of DYRK1A kinase

DYRKs are classified as dual specificity kinases due to their ability to phosphorylate their own Y side chains as well as their substrates on S/T residues. Unlike the regulatory phosphorylation of the activation Y loop residue in MAPK by upstream kinases, co-translational activating autophosphorylation on the second tyrosine in the YxY motif in DYRK1A occurs *in cis* and does not require other kinases or interacting proteins (Himpel et al., 2001; Lochhead et al., 2003; Lochhead et al., 2005). Interestingly, studies using recombinant bacterially expressed proteins showed that mature mammalian DYRK1A can autophosphorylate the Y side chains outside of its kinase domain and is capable of phosphorylating tyrosine residues in an exogenous substrate (Walte et al., 2013). Although physiological relevance of these findings remains to be validated, an important implication of this study was that certain DYRK1A inhibitors have differential effect on cis-autophosphorylation and the S/T activity towards a protein substrate. Furthermore, a DYRK1A-Y321F activation loop mutant, which is commonly used in research due to its diminished ability to phosphorylate the S/T substrates, was shown to display an increased activity towards the tyrosine sites in *cis* and possibly against some external protein substrates (Walte et al., 2013).

Analysis of *in-vitro* phosphorylated synthetic peptide substrates established DYRK1A’s preference for arginine residue in the −2 or −3 position and for a proline at the +1 position (Himpel et al., 2000; Himpel et al., 2001). Indeed, several identified DYRK1A phosphorylation sites fit the R-X(XX)-S/T-P consensus motif, including substrates such as microtubule-associated protein Tau (Ryoo et al., 2007), Caspase 9 (Seifert et al., 2008), the LIN52 subunit of DREAM repressor complex, DNA repair protein RNF169 and other proteins (Table 1) (Litovchick et al., 2011; Guard et al., 2019; Menon et al., 2019; Roewenstrunk et al., 2019). However, using mass spectrometry-based approach for determining linear kinase substrate motifs and X-ray structural analysis of the substrate recognition pocket, it was revealed that DYRK1A is not a strictly proline-directed kinase and can also recognize small hydrophobic residues such as alanine or valine in the +1 position that would match multiple S/T phosphorylation sites (Soundararaja et al., 2013). This finding is supported by compelling reports of DYRK1A phosphorylating protein substrates that lack a recognizable R-X(XX)-S/T-P consensus motif, such as cyclin D1, glycogen synthase and others (reviewed in (Fernandez-Martinez et al., 2015; Kay et al., 2016)) (Table 2). By phosphorylating a wide range of substrates, DYRK1A can be responsible for regulation of multiple signaling pathways impacting cell cycle, transcription, DNA repair, cytoskeleton function, cell metabolism and other processes, which can explain its profound significance for human health.

**TABLE 1:** DYRK1A phosphorylation sites matching the **R-X(XX)-**p**S/T-P** consensus motif.

Protein (Gene)	Site	Sequence	Effect	Ref
Amphiphysin (AMPHI)	S293	PARPRpSPSQTR	Regulation of endothelin binding	Murakami et al. (2006)
Caspase 9 (CASP9)	T125	VLRPEpTPRPVD	Decreased basal activity	Seifert et al. (2008)
CDC23 (CDC23)	S588	PTRRVpSPLNLS	Increased degradation of cyclin B	Recasens et al. (2021)
Dynamin 1 (DNM1)	S857	PSRPEpSPRPPF	Increased Grb2 binding	Huang et al. (2004)
EIF2BE (EIF2B5)	S539	DSRGGpSPQMDD	Priming for GSK3 inhibitory phosphorylation at S535	Woods et al. (2001a)
FKHR (FOXO1)	S329	ISGRLpSPIMTE	Decreased target gene expression; decreased nuclear localization	Woods et al. (2001b), Bhansali et al. (2021)
GSK3β (GSK3B)	T329	PNGRDpTPALFN	Inhibition of kinase activity	Song et al. (2015)
ID2 (ID2)	T27	ISRSKpTPVDDP	Inhibition of ID1-VHL binding leading to HIF1α degradation	Lee et al. (2016a)
LIN52 (LIN52)	S28	KLDRApSPDLWP	DREAM assembly; decreased protein stability	Litovchick et al. (2011)
Munc18-1 (STXBP1)	T479	QLSRWpTPIIKD	Increased Syntaxin 1 and X11α binding	Park et al. (2012)
Parkin (PRKN)	S131	DSRKDpSPPAGS	Decreased E3 ubiquitin ligase activity	Im and Chung (2015)
Presenilin 1 (PSEN1)	T354	GPHRSpTPESRA	Increased γ-secretase activity	Ryu et al. (2010)
RNF169 (RNF169)	S368 S403	PERSVpSPESND DGRVLpSPLIIK	Increased recruitment to the DNA double strand breaks	Menon et al. (2019), Roewenstrunk et al. (2019)
SRSF1 (ASF)	S277 S234 S238	RSRSYpSPRRSR RRSRGpSPRYSP GSPRYpSPRHSR	Increased ASF localization to nuclear speckles; regulation of Tau splicing	Shi et al. (2008)
SRSF7 (9G8)	S217 S223	KSRSPpSPKRSR PKRSRpSPSGSP	Regulation of Tau exon 10 splicing	Ding et al. (2012)
Tau (MAPT)	T212	GSRSRpTPSLPT	Increase fibril formation	Park et al. (2007), Liu et al. (2008)

**TABLE 2:** DYRK1A non-canonical phosphorylation sites.

Protein (Gene)	Site	Sequence	Effect	Ref
Cryptochrome 2 (CRY2)	S558	PSGPApSPKRKL	Priming for GSK3 phosphorylation at S553; decreased protein stability	Kurabayashi et al., 2010
Cyclin D1 (CCND1)	T286 or T288	LACpTPTpDVRDVDI-c	Decreased protein stability	Soppa et al. (2014)
Cyclin D3 (CCND3)	T283	QTSpTPTDVTAIHL-c	Decreased protein stability	Thomp et al. (2015)
GLI1 (GLI1)	S408	LPRAPpSISTVE	Increased transcription of target genes	Ehe et al. (2017)
GluN2A (NMDAR2A)	S1048	THSLKpSPRYLP	Decreased internalization of NMDA receptors	Grau et al. (2014)
Histone H3 (H3C1)	T45 S57	RYRPGpTVALRE RRYQKpSTELLI	Decreased HP1 binding and transcriptional repression	Jang et al. (2014)
HPV16 E7	T5 or T7	n-MHGDpTPpTLHEY	Increased protein stability	Liang et al. (2008)
p27 (CDKN1B)	S10	n-RVSNGpSPSLER	Increased protein stability	Soppa et al. (2014)
p53 (TP53)	S15	VEPPLpSQETFS	Increased transcription of target genes	Park et al. (2010)
RCAN1 (DSCR1)	T192	RRPEYpTPIHLS-c	Increased binding and inhibition of calcineurin	Jung et al. (2011)
RNA PolII (POLR2A) (CTD repeat)	S2 or S5	YpSPTpSPS	Increased elongation activity	Di Vona et al. (2015)
RNF169 (RNF169)	S688	ERRTVpSRRKGS	Increased recruitment to DNA double strand breaks	Roewenstrunk et al. (2019)
SF3B1 (SF3B1)	T434	RKLTApTPTPLG	Unknown	de Graaf et al. (2006)
Sirtuin 1 (SIRT1)	T522	SELPPpTPLHIS	Increased deacetylase activity	Guo et al. (2010)
Synuclein α (SNCA)	S87	VEGAGpSIAAAT	Increased aggregate formation and neuronal cell death	Kim et al. (2006)
STAT3 (STAT3)	S727	IDLPMpSPRTLD	Increased transcription of target genes	Kurabayashi et al., 2015
TRAF3 (TRAF3)	T29	DRSAGpTPVFVP	Decreased ubiquitin ligase activity	Li et al. (2021b)

## 3 DYRK1A in human disease and therapeutic development

### 3.1 Imbalance of DYRK1A contributes to neuropathological conditions

DYRK1A has been extensively studied because of its role in Down syndrome (DS), one of the most universally recognized genetic syndromes with estimated incidence of approximately 1 out of 1,100 live births. Human DYRK1A protein is encoded by the *DYRK1A* gene residing in the DS critical region on chromosome 21 (ch21) (Ohira et al., 1997; Korbel et al., 2009). Trisomy of this region causes multi-organ developmental abnormalities, facial gestalt, intellectual disability, and early onset of Alzheimer-like neurodegeneration (Dierssen, 2012; Park and Chung, 2013). Identification of the genes on ch21 that contribute to DS-associated phenotypes support the involvement of DYRK1A imbalance in the multisystem manifestations of this syndrome, including abnormal neurological development and neurodegeneration (reviewed in (Park et al., 2009; Stringer et al., 2017; Stagni et al., 2018)). This conclusion is supported by the studies with genetic animal models or the chemical inhibition of DYRK1A (Laguna et al., 2013; Blazek et al., 2015; Garcia-Cerro et al., 2017; Garcia-Cerro et al., 2018). The mechanisms that mediate the multitude of pathological consequences of an increased DYRK1A dosage in the brain of DS patients may involve deregulation of the cell cycle (discussed below), impaired neuronal differentiation and cell death, a precocious onset of neurogenesis and the concomitant depletion of the proliferating progenitors, and abnormal neuronal migration, transport, or synaptic function (reviewed in (Tejedor and Hammerle, 2011; Arbones et al., 2019; Kurabayashi et al., 2019; Atas-Ozcan et al., 2021)).

In further support of its essential role in the development of the brain and other organ systems, haploinsufficiency of the *DYRK1A* gene causes a rare autosomal-dominant intellectual developmental disorder (MRD-7 or DYRK1A syndrome, OMIM #614104). This syndrome manifests with characteristic features such as microcephaly, facial dysmorphism, severe intellectual disability, autism spectrum disorder (ASD), speech impairment and seizures (Bronicki et al., 2015; Ji et al., 2015; van Bon et al., 2016; Evers et al., 2017). In mice, homozygous deletion of the *Dyrk1a* gene is embryonic lethal, whereas heterozygous animals have a decreased body and brain mass, specific neurological and behavioral defects, and febrile seizures (Fotaki et al., 2002; Fotaki et al., 2004; Benavides-Piccione et al., 2005; Raveau et al., 2018; Arranz et al., 2019). Animal models have been useful to elucidate some of the mechanisms by which DYRK1A regulates neurodevelopment. In *Xenopus*, embryos with decreased Dyrk1a had a smaller forebrain due to an abnormal cell cycle progression and cell death (Willse et al., 2020). Dyrk1a was also shown to regulate brain growth in mice correlating with changes in gene expression levels (Guedj et al., 2012). A recent study using a conditional brain cortex-specific knockout of *Dyrk1a* in mice, recapitulated several phenotypes of MRD-7 syndrome and revealed a suppression of MAPK and mTOR signaling (Levy et al., 2021). Importantly, pharmacological treatment with insulin-like growth factor-1 (IGF-1) ligand known to activate these pathways rescued microcephaly and neuronal outgrowth defect in this model, suggesting a possible therapeutic strategy for mitigating the deficiencies phenotypes associated with DYRK1A deficiency syndrome.

DYRK1A is also implicated in age-related neurodegeneration including AD and Parkinson’s disease [reviewed in (Arbones et al., 2019; Deboever et al., 2022)]. An early onset AD is one of the common features in DS individuals. The finding of increased DYRK1A expression in the brain of AD patients without DS suggested that DYRK1A is involved in the pathogenesis of neurodegeneration and dementia associated with this disease (Wegiel et al., 2008; Lee et al., 2017). Moreover, DYRK1A has been associated with accumulation of amyloid beta (Aβ) and the neurofibrillary tangles (NFTs) composed of hyperphosphorylated microtubule-associated protein Tau, the main pathological cause of neurotoxicity associated with AD [reviewed in (Fan et al., 2019)]. Together, these results provide a compelling rationale for therapeutically targeting DYRK1A for prevention or alleviation of AD associated neurodegeneration [reviewed in (Stotani et al., 2016; Pathak et al., 2018; de Souza et al., 2023)].

Consistently, several DYRK1A inhibitors have demonstrated promising results in rescuing the Aβ and NFTs phenotypes in various *in vivo* models of AD (Kim et al., 2016; Melchior et al., 2019; Velazquez et al., 2019; Stensen et al., 2021; Zhu et al., 2022; Grygier et al., 2023; Liu et al., 2023). Among these, the most potent inhibitors of DYRK1A from natural sources include β-carboline alkaloid Harmine, the polyphenolic compound in green tea Epigallocatechin-gallate (EGCG), and the marine alkaloid Leucettamine. Furthermore, several promising synthetic small molecule inhibitors have been characterized for potential application in AD, cancer, and type I diabetes [for excellent recent reviews, see (Kumar et al., 2021; Liu T. et al., 2022; Rammohan et al., 2022; de Souza et al., 2023)]. However, existing DYRK1A inhibitors can have off-target effects, possibly due to the similarity of the CMGC domain in other kinases, including DYRK1B that has 85% sequence identity within the kinase domain. This also presents a challenge for the development of the new DYRK1A-specific inhibitor drugs, although several promising drug candidates are already in the pipeline [reviewed in (Stotani et al., 2016; Pathak et al., 2018; Liu T. et al., 2022)). However, promiscuous targeting of two or more CMGC kinases involved in the AD degeneration could actually be beneficial and warrants further investigation (Demuro et al., 2021; Liu T. et al., 2022; Grygier et al., 2023; Liu et al., 2023). For example, one such promising drug is CX-4945 which not only inhibits DYRK1A but also targets casein kinase II and CLK. This drug is a potent inhibitor of DYRK1A *in vitro*, as well as in *Drosophila* and mouse models of DYRK1A overexpression and is currently in clinical trials for treating cancer and COVID-19 (Siddiqui-Jain et al., 2012; Kim et al., 2016; Halloran et al., 2022; Grygier et al., 2023). In addition, a kinome-wide polypharmacological profile study of the four Food and Drug Administration (FDA)-approved inhibitors of poly (ADP-ribose) polymerase (PARP) revealed an unexpected submicromolar activity of niraparib against DYRK1A and DYRK1B (Antolin et al., 2020; Sandhu et al., 2022). However, the implications of this finding for the clinical efficacy and off-target side effect profile for this drug remain to be investigated. So far, EGCG is the only DYRK1A inhibitor that has been investigated in randomized clinical trials in DS. Although there was a significant positive effect on the body mass index, changes in the lipid profile, and restoration of the mitochondrial function in the groups receiving EGCG supplementation compared to placebo, there was no benefit for either cognitive or functional performance (Xicota et al., 2020; Scala et al., 2021; Cieuta-Walti et al., 2022). This could be due to poor pharmacokinetic properties and limited bioavailability of EGCG in the brain, insufficient duration of the treatment, or because the treatment was administered after the structural changes in the DS brain have already occurred (Duchon and Herault, 2016; Atas-Ozcan et al., 2021). Recently, fluorinated derivatives of EGCG with improved inhibitory activity and bioavailability have been reported with encouraging results in the mouse models of inflammation and Parkinson’s disease (Araldi and Hwang, 2023). Additional novel DYRK1A inhibitors have been identified that can induce DYRK1A degradation that can be useful for decreasing the dosage of the expressed protein in AD and DS, but further research is required to evaluate their potential for therapeutic use (Branca et al., 2017; Melchior et al., 2019; Velazquez et al., 2019). Finally, normalization of the DYRK1A function in AD and DS could be achieved by developing the approaches for decreasing the protein levels without targeting its catalytic activity, such as use of PROteolysis Targeting Chimeras (PROTACs) (Choudhary et al., 2023; Thomas et al., 2023).

### 3.2 DYRK1A in cell cycle and cancer

During neurological development, DYRK1A overexpression could decrease the number of neurons generated by each neural progenitor by inducing a premature cell cycle arrest. This effect could be mediated by several key cell-cycle regulators reportedly downstream of DYRK1A, including D-type cyclins, CDK inhibitors p21/CIP1 (encoded by CDKN1A) and p27/KIP1 (encoded by CDKN1B), as well as the LIN52 subunit of the DREAM (DP1, Rb-like, E2F4, and MuvB core) complex, a multi-subunit transcriptional repressor that regulates >800 genes involved in cell division (reviewed in (Fernandez-Martinez et al., 2015; Kay et al., 2016; Wa et al., 2021). Importantly, among these substrates, only the regulatory S28 site in LIN52 appears to be predominantly phosphorylated by DYRK1A or DYRK1B but not any other kinases (Litovchick et al., 2011; Iness et al., 2019; Menon et al., 2019). LIN52 is an integral part of the MuvB core (LIN9, LIN37, LIN52, LIN54 and RBBP4), a stable protein complex that recognizes cell-cycle genes homology (CHR) promoter signature, required for assembly of DREAM (Litovchick et al., 2007; Muller and Engeland, 2009; Schmit et al., 2009; Litovchick et al., 2011). Phosphorylation of LIN52 at S28 by DYRK1A creates a direct binding site for Rb-related p130 protein that is required for the DREAM formation (Guiley et al., 2015). In the S-phase of the cell cycle, LIN52 dissociates from CDK-phosphorylated p130 and mediates assembly of MuvB core with transcription factor B-Myb that is required for mitotic gene expression and cell cycle progression (Schmit et al., 2007; Sadasivam et al., 2012; Sadasivam and DeCaprio, 2013; Guiley et al., 2018; Schade et al., 2019). Interestingly, overexpression of B-Myb in human cells results in accumulation of un-phosphorylated LIN52 and DREAM disassembly similar to the effect of DYRK1A inhibition, without affecting the DYRK1A kinase activity (Iness et al., 2019). Since high expression of B-Myb is oncogenic and serves as clinical marker of poor prognosis in breast and other cancers (Iness and Litovchick, 2018), further mechanistic studies of its effect on the DYRK1A substrate LIN52 are justified.

Depletion or chemical inhibition of DYRK1A results in stabilization of the un-phosphorylated form of LIN52 protein, DREAM disassembly and upregulation of the cell cycle dependent gene expression (Litovchick et al., 2011; Iness et al., 2019; Massey et al., 2021). However, disruption of the DREAM complex alone is not sufficient to drive the cell cycle re-entry of G0-arrested quiescent cells due to the pRb-mediated inhibition of E2F transcription (Mages et al., 2017; Schade et al., 2019). Importantly, inhibition of DYRK1A could drive the quiescent cells into the cell cycle because it also inactivates the Rb family by stabilizing the D-type cyclins and increasing CDK activity (Chen et al., 2013; Soppa et al., 2014; Najas et al., 2015; Thomp et al., 2015; Hille et al., 2016). This model is supported by evidence that DYRK1A inhibition results in increased mouse cardiomyocyte cycling (Hille et al., 2016; Lan et al., 2022; Yo et al., 2022), proliferation of human pancreatic beta cells (Wang P. et al., 2022; Guo et al., 2022; Mullooly et al., 2023), and S-phase re-entry of adult hepatic progenitor cells (Kruitwagen et al., 2018). Furthermore, a recent phosphoproteomic screen uncovered another important pathway of cell cycle control mediated by DYRK1A-dependent phosphorylation of CDC23, a key component of the anaphase-promoting complex ubiquitin ligase that is necessary for degradation of mitotic cyclin B (Recasens et al., 2021). A thorough investigation of the functional relationship between DYRK1A and key cell cycle regulators will be important to validate the DYRK1A targeting as a clinical approach to promote cell cycle re-entry in these critical quiescent cell populations.

Given that the chronic conditions caused by high expression of DYRK1A, such as DS and AD, would require a long-term treatment with DYRK1A inhibitor drugs, it will be essential to evaluate the safety of these drugs given the emerging role of this kinase in cancer. Indeed, both gains and losses of the *DYRK1A* gene are common in adult human cancers, and their effect on survival could be context-specific, according to the data generated by the TCGA Research Network (https://www.cancer.gov/tcga). While a majority of the solid tumor TCGA datasets display a DYRK1A gene copy number loss, several cancers including testicular germ cell cancer, diffuse large B-cell lymphoma and uveal melanoma display high rates of an increased DYRK1A gene dosage. The gene copy number variation data is supported by analysis of the RNA sequencing data, which also indicates a significant downregulation of DYRK1A in most, but not all cancer types (Boni et al., 2020; Laham et al., 2021). Recent data support the targeting of DYRK1A for cancer therapy and to increase the tumor sensitivity to other cancer treatments. In preclinical lung cancer tumor models, inhibition of DYRK1A results in decreased STAT3/EGFR/Met signaling and NSCLC sensitization to EGFR inhibitor AZD9291 (Li YL. et al., 2019). Similarly, DYRK1A inhibition suppressed the EGFR/c-Met signaling in pancreatic cancer, resulting in a decreased tumor growth (Luna et al., 2019; Zhao et al., 2020). In glioblastoma (GBM), DYRK1A was found to be highly expressed in EGFR-amplified tumors, whereas doxycyclin-inducible shRNA depletion of DYRK1A decreased tumor growth in the orthotopic xenograft models (Pallavicini et al., 2019). In the same study, the treatment with DYRK1A inhibitor Harmine as a single agent increased the survival of tumor-bearing animals. Importantly, DYRK1A inhibition promoted the EGFR degradation and decrease of the self-renewal capacity of the primary GBM cells (Pozo et al., 2013). DYRK1A could be especially relevant to the progression of glioblastoma since it has been also shown to control the ID2-HIF2α pathway, whereby the DYRK1A inhibition could block the HIF2α degradation and promote the tumor growth (Lee SB. et al., 2016). The dual role of DYRK1A in cancer could be due to its requirement for maintaining the quiescent or dormant state, which is important for cancer cell survival under the genotoxic stress conditions, such as chemotherapy [for recent review, see (Rammohan et al., 2022)]. At least in some tumor types, such as the primary ovarian cancer spheroids and gastrointestinal stromal tumors (GIST), the pro-survival role of DYRK1A is mediated by promoting the DREAM complex formation and entry into G0/G1 arrest (Boichuk et al., 2013; MacDonald et al., 2017). Furthermore, since the DREAM complex represses the transcription of many genes with function in the DNA damage signaling and repair pathways (Fischer et al., 2015; Lang et al., 2021; Bujarrabal-Dueso et al., 2023), DYRK1A could have an impact on the efficacy of anti-cancer treatments targeting these pathways. Therefore, the role of DYRK1A in the cancer treatment response and resistance needs to be further studied using clinically relevant tumor models and rationally designed drug treatment regimens.

Interestingly, DS individuals across all age groups have a significantly lower risk of dying from all types of cancer, with exception of testicular cancer and DS-specific types of pediatric leukemia (Yang et al., 2002). The role of DS critical region in tumor suppression is strongly supported by studies using animal models, although the contribution of the individual genes, including DYRK1A, remains to be further investigated (Sussan et al., 2008; Reynolds et al., 2010; Yang and Reeves, 2011; Lee et al., 2013; Shin et al., 2014). DYRK1A could contribute to the DS tumor suppression though its role in cellular senescence, a distinct type of cell cycle arrest that prevents the expansion of cells that acquired an oncogenic mutation (Campisi, 2001). In support of this hypothesis is the data showing that inhibition of DYRK1A in immortalized fibroblast cell lines results in a bypass of the oncogenic Ras-induced senescence and increased proliferation (Litovchick et al., 2011). On the other hand, the overexpression of DYRK1A in the DS-specific B-cell acute lymphoblastic leukemia (B-ALL) and acute megakaryoblastic leukemia (AMKL) has been shown to target distinct cellular pathways (Malinge et al., 2012; Lee P. et al., 2016). In B-ALL, inhibition of DYRK1A decreased cell proliferation and induced cell death by disrupting the FOXO1 and STAT3 transcription (Bhansali et al., 2021). Interestingly, conditional knockout of Dyrk1a in mouse lymphoid precursor cells results in reduced number of B- and T-cells by disrupting a critical cell cycle exit step required for differentiation (Thomp et al., 2015). In this model, a Dyrk1a-mediated turnover of cyclin D3 served as a key mechanism for the proper transition from a proliferative state to quiescence. Furthermore, studies using mouse genetic models found that Dyrk1a plays an important role in mature B-cell function in response to infection or vaccination by regulation of class-switch recombination mediated by phosphorylation of mutS homolog 6 (MSH6) protein (Stoler-Barak et al., 2023). MSH6 is a part of a mismatch recognition complex that is mutated in several types of cancer, including the hereditary Lynch syndrome (Abildgaard et al., 2023). These studies emphasize the essential role of DYRK1A in hematopoiesis, immune system function, and hematological malignancies, and justify further studies aimed at better understanding of the dynamics of the molecular interactions of DYRK1A in this system.

Finally, DYRK1A could also modulate cancer risk by interacting with oncogenic viral proteins that target cell cycle regulators, such as Adenovirus E1A protein, high-risk HPV16/18 E7 proteins as well as cutaneous HPV E6 proteins (Liang et al., 2008; Cohen et al., 2013; Kuppuswamy et al., 2013). High risk HPV16 and HPV18 infection is responsible for up to 5% of all human cancers, including cervical and oral carcinoma (McLaughlin-Drubin et al., 2012; Ghittoni et al., 2015). The role of DYRK1A in the HPV16/18 infection and cancer pathogenesis is not well understood and appears to be contrary to its cell cycle control function. Indeed, DYRK1A protein levels are increased in the HPV16-transformed keratinocytes while DYRK1A phosphorylation of HPV16 E7 at T5 and T7 residues leads to its stabilization and increased transforming activity (Chang et al., 2007; Liang et al., 2008). This is especially intriguing because the HPV16/18 E7 proteins are known to displace LIN52 from the DREAM complex by a direct competition for the Rb-like p130 binding (Guiley et al., 2015; Fischer et al., 2017; James et al., 2021). On the other hand, DYRK1A serves as one of the host factors that restrain the transforming activity of Adenovirus 5 E1A protein and low-risk cutaneous HPV E6 (Komorek et al., 2010; Kuppuswamy et al., 2013). Interestingly, although overexpression of DYRK1A inhibits proliferation of many cancer cell lines, the HEK 293T cells that express Simian Virus 40 (SV40) LT antigen and E1A proteins are resistant to this effect. This suggests that the cellular pathways targeted by these viral oncoproteins are essential for DYRK1A-mediated growth suppression (Litovchick et al., 2011). The interaction of DYRK1A with transforming viral proteins could be important for interpretation of the studies performed using cell lines that express such factors, such as HEK293T or HeLa, because of the protein-protein interactions between DYRK1A and host cell proteins that can be mediated by viral factors. Furthermore, DYRK1A could play a broader role in virus-host cell interaction beyond the cell cycle effects, since it has been shown to influence the infection by pathogenic human coronaviruses, HIV and human cytomegalovirus (HCMV) (Hamilton et al., 2018; Kisaka et al., 2020; Wei et al., 2021; Strine et al., 2023). However, our knowledge of the roles of DYRK1A in virus-host cell interaction, viral pathogenesis and virus-driven oncogenic transformation remains incomplete, and the future studies to address these gaps of knowledge are warranted.

## 4 Understanding the function and regulation of DYRK1A through its interactome

### 4.1 Studies of DYRK1A protein interactions

**FIGURE 2:**
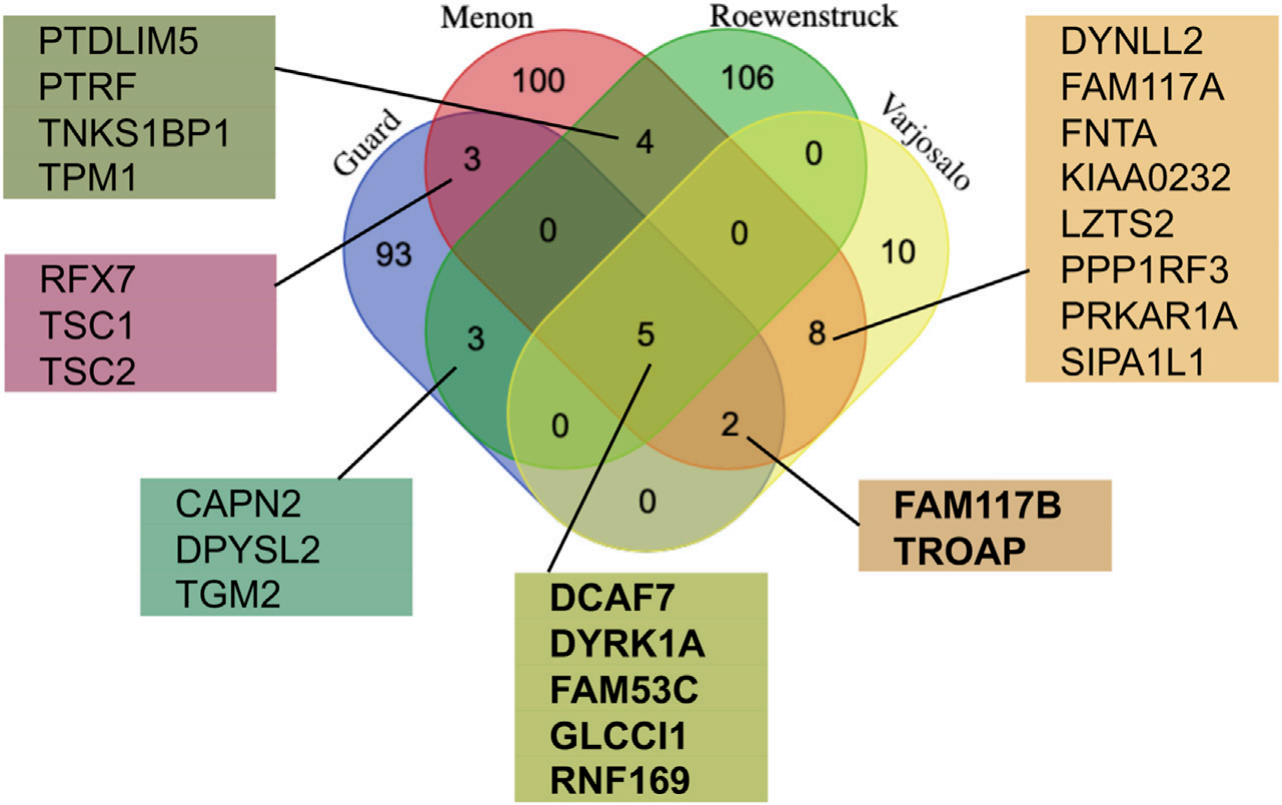
Comparison of the DYRK1A proteomic studies reveals the most reproducible interactors. The lists of the protein hits from the four independent studies using DYRK1A as a bait (Varjosalo et al., 2013; Guard et al., 2019; Menon et al., 2019; Roewenstrunk et al., 2019) were obtained from the BioGrid, and compared using publicly available web tool (http://bioinformatics.psb.ugent.be/cgi-bin/liste/Venn/calculate_venn.htpl). Proteins detected in at least three independent studies (shown in bold) are discussed in detail here.

In an attempt to better understand the function and regulation of DYRK1A, multiple groups have studied the proteomic landscape of its protein-protein interaction network. The BioGrid protein interaction network database currently lists more than 400 unique proteins that interact with human DYRK1A (thebiogrid.org). A mass spectroscopy proteomic screen with several CMGC family kinases expressed in HEK293 cells revealed the distinct interacting protein networks, highlighting their specific roles in the cell (Varjosalo et al., 2013). Not surprisingly, based on their structural similarity, DYRK1A and DYRK1B had the most overlapping interactomes, suggesting that these kinases may play redundant roles in certain cell types that express both proteins, such as skeletal myocytes (Varjosalo et al., 2013; Dong et al., 2021). Further studies characterized the DYRK1A interactomes using either ectopic expression in the human glioblastoma cell line T98G (Menon et al., 2019), or endogenous DYRK1A immunoprecipitated from HeLa cells (Guard et al., 2019; Roewenstrunk et al., 2019). These studies identified more than 300 unique hits specifically detected in DYRK1A immunoprecipitates, including 25 interactors detected in at least two independent studies (Figure 2).

The proteins detected in 3 out of 4 DYRK1A proteomic studies include DYRK1A, DCAF7, FAM53C, FAM117B, GLCCI1, RNF169 and TROAP. Apart from DCAF7 and RNF169, the biochemical and functional characterization of these DYRK1A interacting proteins has not yet been reported. Reproducible detection of a binding partner across independent studies, purification approaches, and different cell lines suggests that these proteins form relatively stable complexes with DYRK1A and could be significant for the function of this kinase. Below is a review of current literature on characterization of these DYRK1A interacting proteins.

### 4.2 DCAF7

DDB1 and CUL4 associated factor 7 (DCAF7, also known as WDR68 or HAN11) is the most enriched DYRK1A binding protein detected in multiple reciprocal proteomic studies (thebiogrid.org), and the most characterized to date. Like *DYRK1A*, the *DCAF7* gene is conserved in evolution and has been shown to play roles in a range of developmental and other essential processes. DCAF7 orthologue YPL247C in *Saccharomyces cerevisiae* yeast cells directly interacts with the founding member of the DYRK sub-family member Yak1 and plays a role in stress response G-protein signaling (Burchett et al., 2002; Breitkreutz et al., 2010). In *C. elegans*, SWAN-1 and SWAN-2, orthologues of DCAF7 are involved in regulation of the osmotic stress response (Ritterhoff et al., 2010). The fruit fly DCAF7 ortholog Wap (wings apart, also known as Riquiqui), is essential for the normal wing-vein patterning and in the development of the adult jump muscle (Morriss et al., 2013). Wap also interacts with the fly Dyrk1a orthologue, Mnb, and both genes are involved in regulation of the Salvador-Warts-Hippo (SWH) pathway (Degoutin et al., 2013).

Dcaf7 has been shown to have a role in craniofacial development in zebrafish, where it regulates the expression of edn1 (endothelin-1) and downstream targets required for jaw cartilage development (Wang et al., 2013; Alvarado et al., 2016). Interestingly, in this model the nuclear access of Dcaf7, that is facilitated by the presence of Dyrk1a, is required for proper jaw cartilage formation. Consistently, both *dcaf7* and *dyrk1a* are expressed in the presumptive craniofacial tissues of another vertebrate developmental model *Xenopus* (Bonano et al., 2018; Blackburn et al., 2019).

In mammalian cells, DCAF7 has been shown to regulate the levels of DYRK1A (Nissen et al., 2006; Wang et al., 2013; Yousefelahiyeh et al., 2018). In fact, both knockdown or overexpression of DCAF7 can cause similar reductions or upregulation not only of DYRK1A but also DYRK1B (Yousefelahiyeh et al., 2018). This regulation does not involve proteasomal degradation or control of mRNA expression, and the mechanism of this effect is not fully understood. Since the DCAF7 binding regions in DYRK1A and DYRK1B, corresponding to amino acids 93–104 of human DYRK1A, are adjacent to a conserved DYRK homology box (DH), the interaction with DCAF7 could be important for the proper folding of the kinase domain (Ritterhoff et al., 2010; Glenewinkel et al., 2016). Given their exquisite connection and reliance on each other, it will be important to investigate whether the dysfunction of DCAF7 is responsible for some of the developmental phenotypes associated with abnormal DYRK1A activity. The findings described above suggest that animal models with distinct, measurable developmental phenotypes, such as craniofacial or wing development, could be valuable for the future characterization of functional and biochemical aspects of the DYRK1A-DCAF7 interaction.

Structurally, DCAF7 is a 39 kDa protein that has a β-propeller shape formed by seven tryptophan-aspartate dipeptide repeats that are 44–60 amino acids in length (WD40 domains), which can facilitate protein-protein interactions (Stirnimann et al., 2010). Based on structural similarity, DCAF7 was predicted to act as a substrate receptor for the DDB1-Cullin ubiquitin ligase complexes (Jin et al., 2006; Lee and DCAFs, 2007). Recent studies identified substrates of DCAF7-DDB1-Cul4, including MEN1 (mutated in neuroendocrine tumors) protein (Xu et al., 2023), and DNA Ligase I involved in DNA replication and base excision repair (Peng et al., 2016). Interestingly, DCAF7 is also involved in maintaining the protein expression levels of the nucleotide excision repair subunits of the ERCC1-ERCC4 endonuclease complex (Kawara et al., 2019), suggesting a broader role of DCAF7 in various DNA repair pathways. Interestingly, mutations in the *ERCC* genes cause a number of rare developmental disorders including cerebrooculofacioskeletal syndrome (COFS), characterized by intellectual disability, microcephaly, and facial abnormalities similar to patients with DYRK1A syndrome (Manandhar et al., 2015).

Importantly, DCAF7 can also act as a scaffold to mediate the interaction between other proteins, such as MEKK1 and DYRK1A kinases, or between E1A and DYRK1 kinases (Ritterhoff et al., 2010; Glenewinkel et al., 2016). In fact, the C-terminal region of E1A, involved in the binding of DYRK1A and DCAF7, is required for oncogenic transformation in cooperation with E1B, while antagonizing the transformation by Ras (Komorek et al., 2010; Cohen et al., 2013; Zemke and Berk, 2017). The mechanism that could explain this paradoxical activity remains to be characterized and could involve the DCAF7 binding with MEKK1, an important relay kinase in the Ras signaling pathway (Ritterhoff et al., 2010; Glenewinkel et al., 2016). Another important pathway that involves DCAF7 is insulin receptor signaling mediated by interaction with a regulatory protein IRS1 (Frendo-Cumbo et al., 2022). Specifically, loss of DCAF7 results in inhibition of AKT1 and nuclear accumulation of FOXO1, resulting in cell cycle arrest in G2 and increased apoptosis. These findings suggest that targeting DCAF7 could be a beneficial treatment of cancers relying on the IRS1/AKT1 pathway. Of note, although DYRK1A was also shown to interact with IRS1 in this study, manipulation of DYRK1A levels did not phenocopy the effects of DCAF7, emphasizing the need for further elucidation of the complexity of these interactions.

The role of DCAF7 in regulation of gene expression has been described in many organisms. It was first noted that the plant orthologue AN11 was required for transcriptional activation of the anthocyanin genes (de Vetten et al., 1997). DCAF7 has been also found to control of DYRK1A-activated GLI1 transcription through the interaction with mDia1 (DIAPH1) (Morita et al., 2006). Recent studies implicate DCAF7 in the regulation of RNA Polymerase II mediated transcription. It does so by recruiting DYRK1A to C-terminal domain (CTD) of RNA Polymerase II, where it phosphorylates the Serine 2 and Serine 5 residues required for its activity (Di Vona et al., 2015; Yu et al., 2019). Although RNA Polymerase II is nearly universally present at the promoters of actively transcribed genes, a chromatin-wide profiling revealed that DYRK1A is targeted to a specific TCTCGCGAGA motif present at the transcriptional start site of a subset of genes (Di Vona et al., 2015). Such genes include those modulated by cell cycle regulators and the components of the protein synthesis machinery which represent approximately 5% of active promoters (Wyrwicz et al., 2007). This finding suggests the presence of a sequence-specific DNA binding factor that could target the DYRK1A-DCAF7 complexes to these promoters. A member of zinc finger protein family ZBTB33 (also known as Kaiso) could be this factor since it has been shown to recognize the DYRK1A targeted sequence motif in human cell lines, in addition to its well-established binding to 5-methylcytosine at mCpG sites in DNA (Nikolova et al., 2020; B et al., 2013). Interestingly, DYRK1A has been shown to play a role in Kaiso-regulated gene expression by promoting the recruitment of p120-catenin of the Wnt pathway to Kaiso-occupied promoters (Hong et al., 2012). Given the significance of ZBTB33/Kaiso function in the neurological processes and cancer (Schackmann et al., 2013; Kulikova and Kulikov, 2018), further investigation of the functional interaction between DCAF7, DYRK1A and Kaiso is justified.

DCAF7 has also been shown to associate with atypical Polycomb Repressor Complex type 1 (PRC1) by an affinity purification mass spectrometry proteomic screen (Hauri et al., 2016). All six known mammalian PRC1 complexes include the RING1A or RING1B E3 ubiquitin ligases responsible for monoubiquitination of histone H2A at lysine 119 (H2AK119ub1) (Buchwald et al., 2006; Gao et al., 2012; Chittock et al., 2017). Interestingly, DCAF7 was associated specifically with PRC1.3 and PRC1.5 (PRC1.3/5) complexes characterized by the presence of AUTS2 (Autism susceptibility candidate 2), FBRS (fibrosin), or FBRSL1 subunits, as well as Casein Kinase II (Hauri et al., 2016). Although the components of the PRC1.3/5 complexes were not detected in the DYRK1A proteomic studies, the possibility of their functional interaction through their common binding partner DCAF7 remains a possibility that could be explored in the future studies.

### 4.3 GLCCI1 and FAM117 family

Glucocorticoid-induced transcript 1 (GLCCI1) protein is a glucocorticoid-induced protein of unknown function. The protein structure predicts a 58 kDa protein with a coiled coil region and multiple putative S/T phosphorylation sites. Genetic polymorphism in the *GLCCI1* gene, including its promoter region, has been associated with glucocorticoid response in asthma patients, pediatric nephrotic syndrome, and Graft-versus-Host disease (Cheong et al., 2012; Chiba et al., 2018; O'Meara et al., 2015). Mechanistically, GLCCI1 promotes the binding between glucocorticoid receptor (GR) and GR-interacting protein 1 (GRIP1) but inhibits the recruitment of GRIP1 to interferon regulatory factors IRF1 and IRF3 (Hu et al., 2021). In a mouse model of asthma GLCCI1 was shown to play a role in airway remodeling by modulating inflammatory signaling (Xun et al., 2021a; Xun et al., 2021b). Interestingly, analysis of morbidity across the lifespan found a decreased risk of asthma in Down syndrome patients compared to general population or patients with other forms of intellectual disability (Baksh et al., 2023). This could point to a possible functional link between GLCCI1 and DYRK1A in asthma, and further characterization of the role of DYRK1A in the GLCCI1 function could potentially lead to new avenues in asthma research.

**FIGURE 3:**
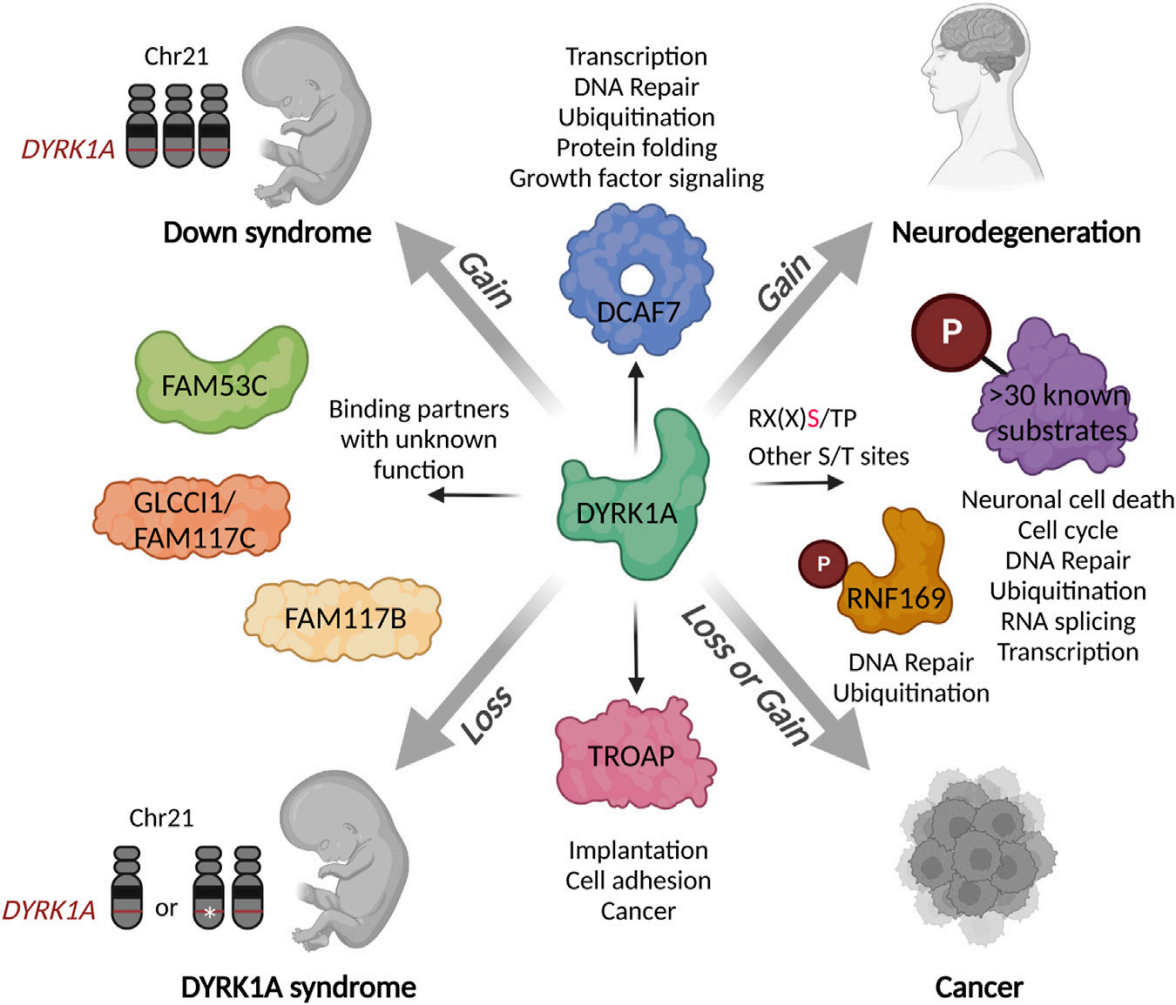
Schematic presentation of the DYRK1A interaction network in the context of human disease. Both gains or losses of DYRK1A during the development or in the adult organism could deregulate various biological processes mediated by multitude of its substrates and binding partners. Characterization of distinct DYRK1A functional networks in specific cell types or stages of development could inform the future therapeutic development for a precise fine-tuning of its activity. Created with http://www.BioRender.com.

Interestingly, GLCCI shares a high sequence similarity with two other DYRK1A-binding proteins FAM117A and FAM117B (Family with sequence similarity 117A and B) (Figure 3). These proteins have not yet been functionally characterized with respect to embryo development or human disease. Genome-wide association studies (GWAS) identified FAM117B as a candidate gene associated with conditions such as intellectual disability (Roberts et al., 2014) or cerebral small vessel disease, while the gene expression profile suggests that it was expressed in astrocytes, neurons, and oligodendrocytes (Chung et al., 2019). Limited studies found contradictory roles of FAM117A and FAM117B in cancer. For example, a bioinformatic analysis of cancer transcriptomes revealed that low levels of FAM117A correlated with a poor prognosis in lung adenocarcinoma. This was confirmed in functional studies demonstrating that FAM117A suppresses cell proliferation in human cancer cell lines (Wu et al., 2022). On the other hand, FAM117B was highly expressed in gastric cancer and promoted tumorigenesis by deregulating the KEAP1/NRF2 signaling pathway that activates transcriptional program in response to oxidative stress (Zhou et al., 2023). Given strong association of the FAM117 family proteins with DYRK1A, their role as downstream effectors or possibly the regulators of DYRK1A function needs to be further investigated using the appropriate animal models of neurological development and cancer.

### 4.4 RNF169

RNF169 is a 77 kDa protein that contains an N-terminal RING finger motif, a central MIU1 and a C-terminal MIU2 (motif interacting with ubiquitin) domains that recognizes the ubiquitinated histone marks and accumulates near the DNA double strand break (DSB) lesions (Chen et al., 2012; Panier et al., 2012; Poulsen et al., 2012). The role of RNF169 in development and human disease has not been extensively investigated; it was suggested that that high expression of RNF169 could be a prognostic marker in pancreatic ductal carcinoma although this association has not been experimentally validated yet (Wang J. et al., 2022). Due to its lack of enzymatic activity, RNF169 antagonizes the function of its close homologue RNF168 an active ubiquitin ligase that recognizes the same K13/K15-ubiquitinated H2A and amplifies the DNA damage response by further ubiquitinating its substrates at site of the lesion (Hu et al., 2017; Kitevski-LeBlanc et al., 2017; An et al., 2018). Loss of RNF169 results in excessive accumulation of 53BP1 protein that blocks the homologous recombination (HR) DSB repair (Poulsen et al., 2012; An et al., 2018). Overexpression of RNF169 thereby expels 53BP1 and promotes the cells to favor HR repair instead of the error-prone non-homologous end joining (NHEJ) (An et al., 2018). Several studies independently identified RNF169 as a binding partner and a substrate of DYRK1A and described a role of DYRK1A in regulation of the RNF169 function, 53BP1 accumulation and the DSB repair (Guard et al., 2019; Menon et al., 2019; Roewenstrunk et al., 2019). The mechanistic role of DYRK1A in the DNA repair and cell survival remains to be further clarified, since these studies found either decreased or increased sensitivity to ionizing radiation in the cells depleted of DYRK1A. Thus, the mechanisms and the factors that govern the role of DYRK1A in DNA repair need to be investigated in the context of cell cycle, considering the role DREAM complex as the master repressor of the DNA damage response transcription program (Fischer et al., 2015; Menon et al., 2019; Bujarrabal-Dueso et al., 2023; Kandhaya-Pillai et al., 2023). Such factors could include a multifunctional ubiquitin specific protease USP7 that is required for nuclear localization of RNF169 (An et al., 2017). USP7 has been shown to interact with DYRK1A in a proteomic study (Menon et al., 2019), and further studies could reveal more details about the functional relationship between these proteins.

### 4.5 TROAP

The *TROAP* gene encodes Tastin, a protein that forms a complex with trophinin and bystin, hence it was named the TROphinin Associated Protein. The TROAP-trophonin-bystin complex is required for the initial adhesion of the blastocyst to uterine epithelial cells at the time of embryo implantation (Fukuda and Nozawa, 1999). Expression of TROAP is developmentally restricted, and is absent in most adult tissues except testis, bone marrow and thymus. However, high levels of TROAP have been reported in several cancer types, including serous ovarian carcinoma, suggesting its utility as a marker for cancer progression and prognosis (Partheen et al., 2008; Godoy et al., 2013; Hu et al., 2019; Liu H. et al., 2022; Li et al., 2022). In mammalian cells, the TROAP protein is upregulated in the G2/M phase of the cell cycle when it associates with microtubules to maintain bipolar spindle and the integrity of the centrosome during mitosis (Nadano et al., 2002; Yang et al., 2008).

The role of TROAP in cancer has been extensively studied and could be different depending on the type and stage of the disease. While it was found to be pro-tumorigenic and associated with poor prognosis in most cancer types, including glioma, lung, breast, ovarian, colorectal, gastric and prostate cancer (Li K. et al., 2019; Jin et al., 2020; Zhao et al., 2021; Liu H. et al., 2022; Li et al., 2022; Wang et al., 2023; Yue et al., 2023), some studies suggest that high expression of TROAP could predict better survival in acute myeloid leukemia (He et al., 2023). The tumor-promoting role of TROAP has been experimentally validated in several preclinical cancer models, including breast, kidney, liver, and prostate cancer (Li K. et al., 2019; Jin et al., 2020; Li L. et al., 2021; Wang et al., 2023; Yue et al., 2023). Mechanistically, the depletion of TROAP was found to cause cell cycle arrest and inhibition of multiple cancer growth-promoting pathways. In one study, TROAP overexpression resulted in the cytoplasmic retention of DYRK1A and DYRK1B in a hepatocellular carcinoma cell line (Li L. et al., 2021). These findings suggest that TROAP could be a therapeutic target in certain cancer types and provide the rationale for further research into the functional relationship between TROAP and DYRK1A.

### 4.6 FAM53C

FAM53C is a protein with predicted molecular wight of 43kDa, which is encoded within a 5q region on chromosome 5 that is frequently deleted in acute myeloid leukemia or a myelodysplastic syndrome (Lai et al., 2000). While the function of FAM53C is not yet known, another member of this family FAM53B/Simplet has been shown to promote cell proliferation during the embryo development and regeneration in the aquatic vertebrate animal models (Thermes et al., 2006; Kizil et al., 2009). Depletion of simplet in zebrafish embryos resulted in abnormal skeletal development due to a decreased nuclear localization and transcriptional activity of β-catenin, and a similar functional role in regulation of the Wnt pathway was also observed for human FAM53B (Kizil et al., 2014). Interestingly, one of the DYRK1A mass spec proteomic studies detected the FAM53B binding to DYRK1A (Menon et al., 2019), suggesting that this protein family could play a role in the DYRK1A signaling and developmental phenotypes.

## 5 Discussion

Recent studies revealed a striking complexity and diversity of the DYRK1A interacting network in human cells. DYRK1A appears to bind tightly to several proteins with poorly understood physiological and molecular functions, some of which have already been characterized as substrates of this protein kinase. Perturbation of DYRK1A expression levels and activity could have a profound effect on these protein interaction networks, affecting multiple signaling pathways, some of which could be cell-type specific or developmentally restricted. Therefore, there is a rationale for performing the DYRK1A-targeted proteome studies under the physiological and pathological conditions at the relevant developmental stages. Future studies will dig deeper into the DYRK1A protein-protein interactions and dissect their individual roles in the pathological phenotypes caused by an abnormal gene dosage or a deregulated expression of the *DYRK1A* gene. The role of DCAF7 is especially intriguing, and the more thorough investigations of its complex with DYRK1A and its role as a scaffolding factor to mediate the binding with other proteins, are warranted. Further development of animal models to understand the role of the DYRK1A interacting proteins and substrates will be especially valuable to increase our understanding of the DYRK1A-associated phenotypes. Moreover, such studies will help to identify effective therapeutic approaches to treat neurodegenerative disease in DS and AD, as well as to harness the dependency of some cancer cells on DYRK1A.
